# Molecular and phenotypic characterization of *Streptococcus pneumoniae* isolates in a Japanese tertiary care hospital

**DOI:** 10.3389/fcimb.2024.1391879

**Published:** 2024-07-22

**Authors:** Takumi Nakao, Kosuke Kosai, Norihiko Akamatsu, Kenji Ota, Fujiko Mitsumoto-Kaseida, Hiroo Hasegawa, Koichi Izumikawa, Hiroshi Mukae, Katsunori Yanagihara

**Affiliations:** ^1^ Department of Laboratory Medicine, Nagasaki University Graduate School of Biomedical Sciences, Nagasaki, Japan; ^2^ Department of Laboratory Medicine, Nagasaki University Hospital, Nagasaki, Japan; ^3^ Department of Infectious Diseases, Nagasaki University Graduate School of Biomedical Sciences, Nagasaki, Japan; ^4^ Department of Respiratory Medicine, Nagasaki University Graduate School of Biomedical Sciences, Nagasaki, Japan

**Keywords:** macrolide resistance, solithromycin, lascufloxacin, capsular type, sequence type, QRDR

## Abstract

This study aimed to investigate the bacterial characteristics of pneumococcal isolates obtained from a tertiary care hospital in Japan. We analyzed the antimicrobial susceptibility, possession of macrolide resistance genes, pneumococcal serogroup/serotype, and sequence type (ST) of pneumococcal isolates from patients aged 15 years or older between 2011 and 2020 at Nagasaki University Hospital. Of the 73 isolates analyzed, 86.3% showed resistance to macrolides, and 28.8%, 46.6%, and 11.0% harbored *mefA*, *ermB*, and both, respectively. Of the isolates possessing *ermB*, 97.6% showed high levels of macrolide resistance [minimal inhibitory concentration (MIC) range, > 16 µg/mL]. Solithromycin (MIC range, 0.03–0.25 µg/mL), regardless of the presence of macrolide resistance genes, and lascufloxacin (MIC range, 0.06–0.5 µg/mL) showed potent *in vitro* activity against pneumococci. Serotype 19A was the most prevalent (six isolates), followed by serotypes 10A, 15A, and 15B/C (five isolates each). Four serotypes (11A, 19A, 22F, and 23B) and five STs (36, 99, 433, 558, and 3111) were significantly correlated with the presence of macrolide resistance genes. All four isolates with serotype 11A/ST99 and three isolates with serotype 19A/ST3111 harbored both *mefA* and *ermB*. No macrolide resistance genes were detected in either of the two isolates with serotype 22F/ST433, while all ten isolates with serogroup 15 (serotypes 15A and 15B/C, five isolates each) possessed *ermB* alone. Our study revealed the bacterial characteristics of the pneumococcal isolates obtained from our hospital. *In vitro* activity of solithromycin and lascufloxacin against these isolates was confirmed.

## Introduction

*Streptococcus pneumoniae* is a leading cause of community-acquired pneumonia (CAP) and nursing and healthcare-associated pneumonia (NHCAP) and causes invasive pneumococcal diseases (IPDs) such as bacteremia and meningitis (The JRS, 2017). Implementation of pneumococcal conjugate vaccine (PCV) has reduced CAP and IPD caused by vaccine preventable serotype (Bonten et al., 2015). However, it was observed that the proportions of vaccine serotypes decreased, but those of non-vaccine serotypes increased after PCV introduction (Hanquet et al., 2022). Additionally, the resistance to commonly used antimicrobials, including penicillin, macrolides, and fluoroquinolones is of great concern (Li et al., 2023). High rates of macrolide resistance have been reported in Japan and other Asian countries (Kawaguchiya et al., 2020; Li et al., 2023). Macrolide resistance is mediated by several mechanisms such as methylation of the ribosomal macrolide target site and macrolide efflux (Wierzbowski et al., 2007). The *ermB* gene, one erythromycin ribosomal methylase (*erm*) family gene, is a common determinant of macrolide resistance in pneumococci (Schroeder and Stephens, 2016). Furthermore, the efflux mechanisms encoded by the macrolide efflux (*mef*) genes cause resistance to only 14- and 15-membered macrolides (Littauer et al., 2005).

Previous studies have reported relationships between the presence of macrolide resistance genes, capsular typing, and sequence type (ST). In a study conducted in Korea, most strains with serotypes 19F (77.2%) and 19A (87.5%) possessed both *mefA* and *ermB* (Bae and Lee, 2009). In a report from Taiwan, of the azithromycin-resistant isolates carrying both *mefA* and *ermB*, 64% and 24% belonged to serotypes 19F and 19A, respectively, and 33% were ST320, 32% were ST236, and 12% were ST271 (Safari et al., 2014). In a study conducted for Japanese children with IPD between 2010 and 2013, the proportions of macrolide-resistant isolates harboring *mefA* or *ermB* (or both) decreased in vaccine serotypes after PCV7 introduction, whereas the rates of macrolide-resistant isolates mediated by *ermB* in the non-vaccine serotypes 15A, 15B, 15C, and 24 increased (Chiba et al., 2014). A study conducted for isolates from adult patients between 2008 and 2016 in Aichi, located in the middle of Japan, reported that 100% of isolates with serotypes 14, 15A, and 23A harbored *ermB* alone and 89.7% of those with serotype 19F possessed *mefA* alone, while 73.0% of those with serotype 23F harbored both *mefA* and *ermB* (Furuya et al., 2017). In addition, a high prevalence (> 90%) for *ermB* was observed in serotypes 15A, 23A, 15B, 15C, 33F, and 12F and the prevalence for both *mefA/E* and *ermB* was highest in serotype 11A/11D (53.8%), according to a study conducted between 2018 and 2019 in Hokkaido, located in northern Japan (Kawaguchiya et al., 2020). However, these relationships have not been fully investigated in our region, located in western Japan.

Solithromycin, a novel fluoroketolide, and lascufloxacin, a novel fluoroquinolone are two antimicrobial agents with potent activity against *S. pneumoniae*. Following development, both these antibiotics are expected to become treatment options against pneumococcal pneumonia. A study reported that solithromycin was very active [minimal inhibitory concentration (MIC) range, ≤ 0.001–1 µg/mL; MIC_90_, 0.25 µg/mL] against 996 pneumococcal isolates collected from North America, Europe, Asia-Pacific, and other regions (Hawser et al., 2017).

Fluoroquinolones inhibit DNA synthesis by binding to DNA gyrases (GyrA and GyrB) and topoisomerase IV (ParC and ParE) in *S. pneumoniae*. One of the main causes of fluoroquinolone resistance is the gradual accumulation of mutations in ParC and GyrA (Cornick and Bentley, 2012). Lascufloxacin has been reported to show *in vitro* activity against pneumococci with GyrA, ParC, and both mutations (Kishii et al., 2017). It also exhibits lower selectivity of resistant strains after drug exposure to strains with either GyrA or ParC mutation (first-step mutants to fluoroquinolone resistance), compared to levofloxacin and garenoxacin (Murata et al., 2018). In addition, a study reported that lascufloxacin exhibited excellent antibacterial activity against penicillin-susceptible and resistant *S. pneumoniae* isolates (MIC range, 0.03–0.06 µg/mL; MIC_90_, 0.06 µg/mL) collected in Japanese hospitals (Kishii et al., 2017).

This study aimed to investigate the bacterial characteristics of pneumococcal isolates obtained from our hospital, including their susceptibility to a novel fluoroketolide and fluoroquinolone.

## Materials and methods

### Study design

This retrospective study was conducted using pneumococcal isolates obtained from the Nagasaki University Hospital, a tertiary care hospital, between 2011 and 2020. We extracted pneumococcal isolates from respiratory specimens and blood of patients aged 15 years or older from our clinical database. Only the first pneumococcal isolate from individual patients was selected for analysis during the study period. Pneumonia was defined as the presence of new infiltrate on chest radiograph or computed tomography image along with the presentation of clinical symptoms. Isolates from respiratory specimens and blood of patients with pneumonia were stored at −80°C in Microbank or at room temperature after freeze drying. In addition, we listed isolates from respiratory specimens, which were collected from patients without pneumonia and evaluated to be purulent, according to the Miller and Jones classification (Miller, 1963). Of these, we used the isolates stored at −80°C in Microbank for further analysis.

Clinical features, including baseline characteristics, antimicrobial therapy, and outcomes, were collected from the medical records. Pneumonia was classified into three categories, such as CAP, NHCAP, and hospital-acquired pneumonia (HAP), according to the definition proposed by the Japanese Respiratory Society (The JRS, 2017). The severity of pneumonia was assessed using A-DROP, a five-factor scoring system for community-acquired pneumonia (age, dehydration, respiratory failure, orientation disturbances, and low blood pressure), which is a modified version of the CURB-65 and was proposed by the Japanese Respiratory Society (Kosai et al., 2014; The JRS, 2017). The study protocol, including the waiver of informed consent, was approved by the Institutional Review Board of Nagasaki University Hospital (approval number:21091310).

### Antimicrobial susceptibility testing

Antimicrobial susceptibility testing (AST) was performed using a MIC plate customized by Eiken Chemical Co., Ltd. The prepared MIC plate was incubated in 5% CO_2_ at 35 ± 2°C for 20–24 h. The results were interpreted according to CLSI document M100-ED33.

### Identification of *S. pneumoniae* and detection of macrolide resistance genes

DNA was extracted from the bacteria using the boiling method as previously described (Yamakawa et al., 2019; Kikuchi et al., 2023), with minor modifications. Multiplex PCR for *mefA*, *ermB*, and *lytA* for *S. pneumoniae* identification was performed using a QIAGEN Multiplex PCR Kit (QIAGEN) under the following conditions: 15 min at 95°C, 30 cycles of 15 s at 94°C, 15 s at 53°C, and 15 s at 72°C, and 10 min at 72°C. The primers used (NIID, 2021) were shown in **Supplementary Table 1**.

### Multilocus sequence typing

Multilocus sequence typing (MLST) was performed targeting seven housekeeping genes (*aroE*, *gdh*, *gki*, *recP*, *spi*, *xpt*, and *ddl*). PCR for each housekeeping gene was performed using TaKaRa Ex Taq DNA Polymerase (Takara Bio Inc.) and the primers described in PubMLST website (https://pubmlst.org/organisms/streptococcus-pneumoniae/primers) or CDC website (https://www.cdc.gov/streplab/pneumococcus/resources.html) under the following conditions: 2 min at 94°C, 30 cycles of 10 s at 98°C, 15 s at 55°C, and 30 s at 68°C, and 5 min at 72°C. The PCR products were purified using ExoSAP-IT PCR Product Cleanup Reagent (Applied Biosystems). Sequencing reactions were performed using a BigDye Terminator v3.1 Cycle Sequencing Kit (Applied Biosystems). The products were purified using a BigDye Xterminator Purification Kit (Applied Biosystems) and analyzed using a SeqStudio Genetic Analyzer (Applied Biosystems). Allele sequences and STs were determined using the PubMLST database (https://pubmlst.org/organisms/streptococcus-pneumoniae).

### Detection of quinolone resistance-determining region mutations

Quinolone resistance-determining region (QRDR) mutations were detected by Sanger sequencing. The primers used (Pan et al., 1996) were shown in **Supplementary Table 1**. PCR was performed using TaKaRa Ex Taq DNA Polymerase (Takara Bio Inc.) under the following conditions: 5 min at 94°C, 30 cycles of 40 s at 94°C, 40 s at 55°C, and 1 min at 72°C, and 5 min at 72°C (Roychoudhury et al., 2016). The subsequent procedures were as described in MLST. The QRDR nucleotide and amino acid sequences were compared to those of *S. pneumoniae* R6 using the National Center for Biotechnology Information Blast program (https://blast.ncbi.nlm.nih.gov/Blast.cgi).

### Determination of pneumococcal serogroup/serotype

The pneumococcal serogroup/serotype was determined by the latex agglutination method using the ImmuLex Pneumotest Kit (SSI Diagnostica), according to the manufacturer’s instructions with minor modifications. Briefly, a drop (approximately 10 µL) of ImmuLex reagent and the prepared bacterial suspension were applied separately next to each other onto the reaction card. They were mixed using a mixing stick and spread over a circular area on the card. Agglutination was determined after the cards were rocked slowly. Furthermore, additional serotyping was performed for serotypes classifiable by PCR. Briefly, the PCR assays were carried out using QIAGEN Multiplex PCR Kit (QIAGEN) and the primers described in the literature (Pai et al., 2006; Carvalho Mda et al., 2009; Pimenta et al., 2009; da Gloria Carvalho et al., 2010) or CDC website (https://www.cdc.gov/streplab/pneumococcus/resources.html) under the following conditions: 15 min at 95°C, 35 cycles of 30 s at 94°C, 90 s at 54°C, and 60 s at 72°C, and 10 min at 72°C (da Gloria Carvalho et al., 2010).

### Statistical analysis

Numerical or categorical variables were analyzed using the Kruskal-Wallis test or Fisher’s exact test among groups. GraphPad Prism 10 (GraphPad Software) was used for the analyses, and the results were considered statistically significant at p < 0.05.

## Results

### Bacterial characteristics of pneumococcal isolates

A total of 86 patients with pneumonia, aged 15 years and older, were confirmed during the study period and 41 isolates (32 in Microbank and 9 freeze-dried isolates) were available as isolates from patients with pneumonia. These isolates were obtained from 34 sputum, six tracheal aspirates, and one blood. In addition, 32 isolates from purulent respiratory specimens (24 sputum, seven tracheal aspirates, and one aspirate obtained using bronchoscopy), which were collected from patients without pneumonia, were available. In total, 73 isolates were included in this study.

**Figure 1:**
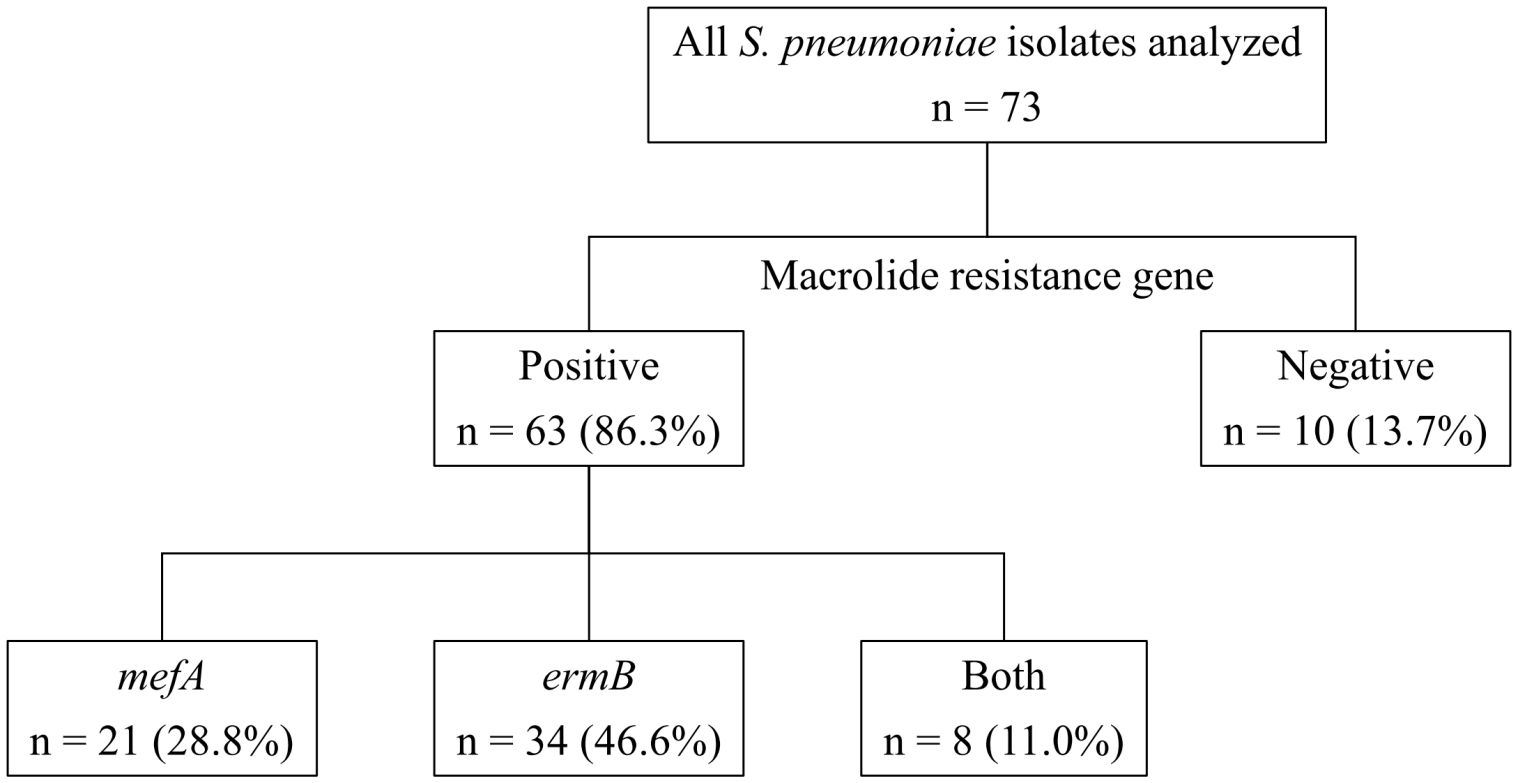
Presence of macrolide resistance genes in pneumococcal isolates.

**Figure 2:**
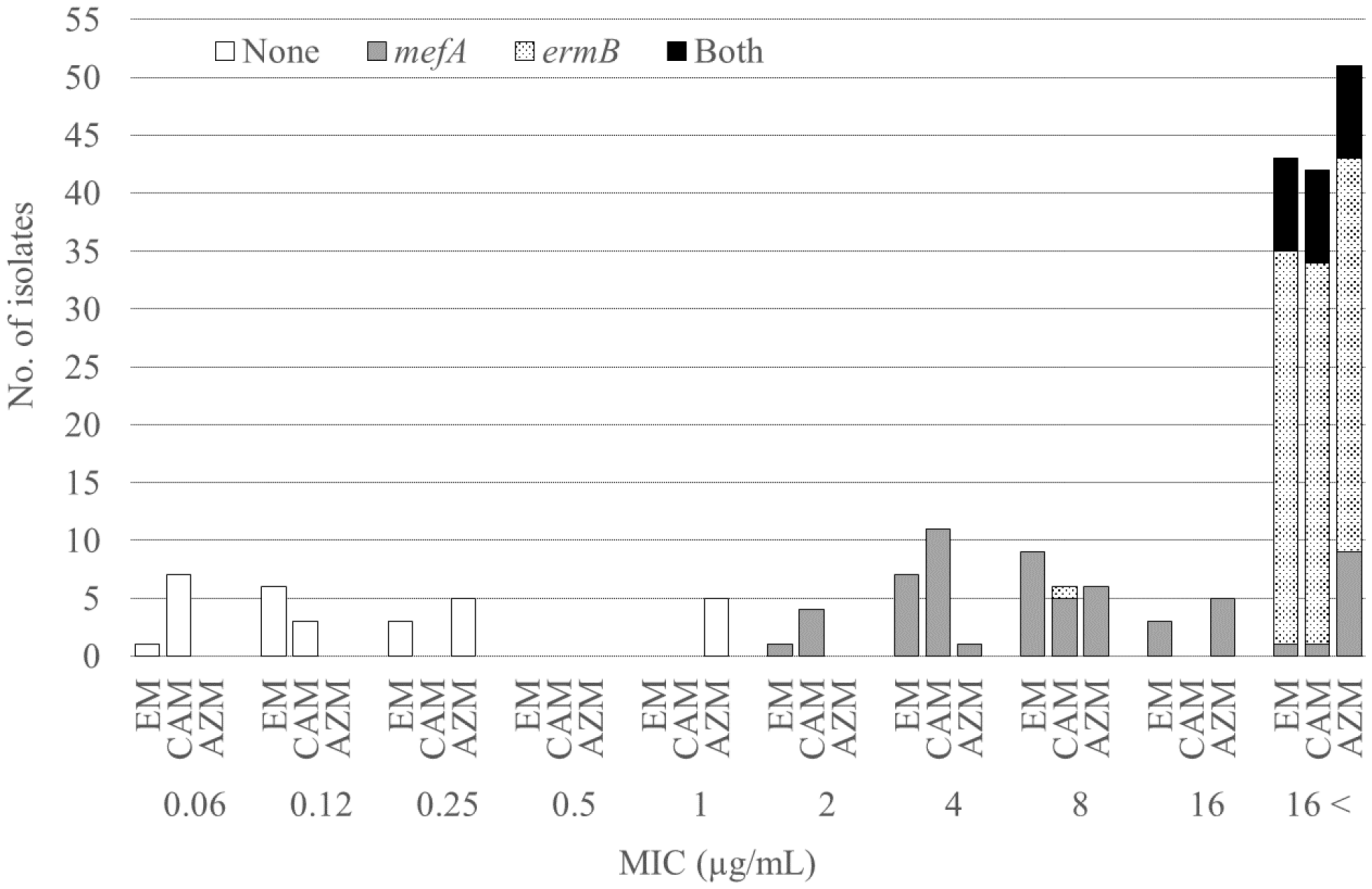
Relationship between the macrolide MICs and the presence of macrolide resistance genes. MIC, minimum inhibitory concentration; EM, erythromycin; CAM, clarithromycin; AZM, azithromycin.

**Figure 3:**
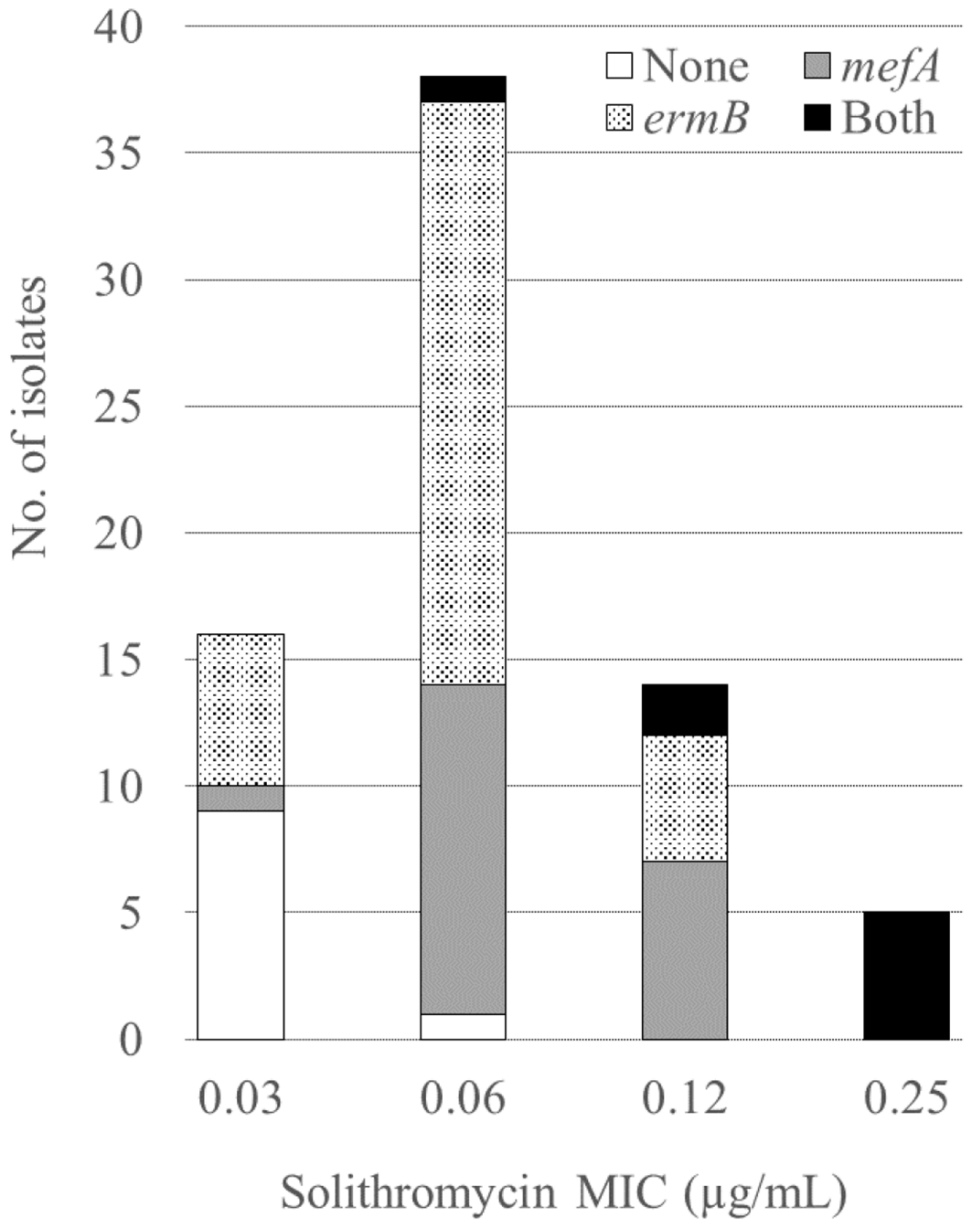
Relationship between the solithromycin MICs and the presence of macrolide resistance genes. MIC, minimum inhibitory concentration.

**Table 1** shows the MICs for the antimicrobial agents, while **Figure 1** shows the presence of macrolide resistance genes in the 73 pneumococcal isolates. The MIC_90_s for all three macrolides (14-membered, erythromycin and clarithromycin; 15-membered, azithromycin) were > 16 µg/mL. Of the 73 isolates, 63 (86.3%) harbored either *mefA* or *ermB* and were resistant to all three macrolides. Of the 73 isolates, 21 (28.8%), 34 (46.6%), and eight (11.0%) tested positive for *mefA*, *ermB*, and both, respectively. The relationship between macrolide MIC and the presence of macrolide resistance genes is shown in **Figure 2**. Among the 42 isolates possessing *ermB*, 41 (97.6%) (33 isolates with *ermB* alone and eight with both *mefA* and *ermB*) showed higher MICs (> 16 µg/mL) for all three macrolides. In contrast, for isolates harboring *mefA* alone, the MICs were distributed 2–> 16 µg/mL for erythromycin and clarithromycin and 4–> 16 µg/mL for azithromycin. High MIC elevation (> 16 µg/mL) for three macrolides were significantly correlated with the presence of macrolide resistance genes (p < 0.0001) (**Table 2**). The MICs for solithromycin ranged 0.03–0.25 µg/mL regardless of the presence of macrolide resistance genes and tended to be lower than those for macrolides (**Table 1**). Meanwhile, although the MICs for solithromycin ranged 0.03–0.06 µg/mL for isolates without *mefA* and *ermB*, this range was wider and higher (0.06–0.25 µg/mL) for isolates harboring both the genes (**Figure 3**). The highest MIC (0.25 µg/mL) for solithromycin were significantly correlated with the presence of macrolide resistance genes (**Table 2**).

**Table 1:** Antimicrobial susceptibility of 73 pneumococcal isolates.

Antimicrobial agent	Minimum inhibitory concentration (µg/mL)		
	Range	50%	90%
Penicillin G	≤ 0.015–8	0.12	1
Erythromycin	0.06–> 16	> 16	> 16
Clarithromycin	0.06–> 16	> 16	> 16
Azithromycin	0.25–> 16	> 16	> 16
Solithromycin	0.03–0.25	0.06	0.12
Levofloxacin	0.5–> 16	1	2
Moxifloxacin	0.12–8	0.25	0.25
Lascufloxacin	0.06–0.5	0.06	0.12

**Table 2:** Relationship between MIC elevation and the presence of macrolide resistance genes in 73 pneumococcal isolates.

Variable	None (n = 10)	*mefA* (n = 21)	*ermB* (n = 34)	Both (n = 8)	P
MIC, > 16 µg/mL					
Erythromycin	0 (0.0)	1 (4.8)	34 (100.0)	8 (100.0)	< 0.0001
Clarithromycin	0 (0.0)	1 (4.8)	33 (97.1)	8 (100.0)	< 0.0001
Azithromycin	0 (0.0)	9 (42.9)	34 (100.0)	8 (100.0)	< 0.0001
MIC, 0.25 µg/mL					
Solithromycin	0 (0.0)	0 (0.0)	0 (0.0)	5 (62.5)	< 0.0001

Data expressed as numbers (column percentages).

MIC, minimum inhibitory concentration.

The MICs for lascufloxacin ranged 0.06–0.5 µg/mL, which showed lower tendency, compared to those for levofloxacin (0.5–> 16 µg/mL) and moxifloxacin (0.12–8 µg/mL). Resistance to levofloxacin and moxifloxacin was observed in two isolates (2.7%). QRDR mutation analysis detected substitutions of S79Y and K137N in ParC and E85K in GyrA in one isolate (MICs, > 16 µg/mL for levofloxacin and 8 µg/mL for moxifloxacin) and substitutions of S79F and K137N in ParC and S81F in GyrA in the other (MICs, 16 µg/mL for levofloxacin and 4 µg/mL for moxifloxacin). The MICs for lascufloxacin against levofloxacin- and moxifloxacin-resistant isolates were 0.5 µg/mL and those for solithromycin were 0.03 or 0.12 µg/mL. Of the 73 isolates, only one (1.4%) showed penicillin resistance.

**Table 3** shows the serogroup/serotype and ST of the pneumococcal isolates classified according to the presence of macrolide resistance genes. **Table 4** shows the relationships between serogroup/serotype or ST and the presence of macrolide resistance genes. Among the isolates identified as serotypes, serotype 19A was the most prevalent (six isolates), followed by serotypes 10A, 15A, and 15B/C (five isolates each), and serotypes 11A, 19A, 22F, and 23B were significantly correlated with the presence of macrolide resistance genes (p < 0.05, respectively) (**Table 4**). Of the isolates showing significant correlations between serotypes and the presence of macrolide resistance genes, all four isolates with serotype 11A harbored both macrolide resistance genes, whereas all two isolates with serotype 22F harbored no macrolide resistance gene. Of the six isolates with serotype 19A, three (50.0%) each harbored *mefA* alone or both *mefA* and *ermB*. Among the four isolates with serotype 23B, three (75.0%) and one (25.0%) possessed no macrolide resistance genes and *ermB* alone, respectively.

**Table 3:** Serogroup/serotype and sequence type of 73 pneumococcal isolates.

Macrolide resistance gene	Serogroup/serotype	Sequence type
None (10)	18 (1)	3594 (1)
	19F (1)	ND (1)
	22F (2)	433 (2)
	23B (3)	36 (2), 355 (1)
	24/31/40 (1)	11184 (1)
	29/34/35/42/47 (1)	558 (1)
	ND (1)	447 (1)
*mefA* (21)	6A/B (1)	282 (1)
	6C (1)	282 (1)
	19A (3)	2331 (3)
	19F (3)	236 (2), 6686 (1)
	19 (non-19A/F) (1)	282 (1)
	16/36/37 (1)	3117 (1)
	21 (1)	1233 (1)
	29/34/35/42/47 (10)	156 (2), 558 (6), 3116 (1), 13347 (1)
*ermB* (34)	3 (3)	180 (2), ND (1)
	6A/B (3)	90 (2), 3787 (1)
	6C (1)	6183 (1)
	7C (1)	2758 (1)
	9V/A (1)	166 (1)
	10A (5)	5236 (5)
	15A (5)	63 (3), ND (2)
	15B/C (5)	63 (3), 199 (1), 2771 (1)
	23A (2)	338 (1), 5242 (1)
	23B (1)	9396 (1)
	13/28 (2)	338 (2)
	24/31/40 (2)	162 (2)
	29/34/35/42/47 (3)	2755 (3)
Both (8)	11A (4)	99 (4)
	19A (3)	3111 (3)
	33F (1)	717 (1)

Values in parentheses indicate numbers of isolates.

ND, not determined.

**Table 4:** Relationship between serogroup/serotype or sequence type and the presence of macrolide resistance genes.

Variable	None	*mefA*	*ermB*	Both	P
Serogroup/serotype^a^					
3 (n = 3)	0 (0.0)	0 (0.0)	3 (100.0)	0 (0.0)	0.5810
6A/B (n = 4)	0 (0.0)	1 (25.0)	3 (75.0)	0 (0.0)	> 0.9999
6C (n = 2)	0 (0.0)	1 (50.0)	1 (50.0)	0 (0.0)	> 0.9999
10A (n = 5)	0 (0.0)	0 (0.0)	5 (100.0)	0 (0.0)	0.1890
11A (n = 4)	0 (0.0)	0 (0.0)	0 (0.0)	4 (100.0)	< 0.0001
15A (n = 5)	0 (0.0)	0 (0.0)	5 (100.0)	0 (0.0)	0.1890
15B/C (n = 5)	0 (0.0)	0 (0.0)	5 (100.0)	0 (0.0)	0.1890
19A (n = 6)	0 (0.0)	3 (50.0)	0 (0.0)	3 (50.0)	0.0033
19F (n = 4)	1 (25.0)	3 (75.0)	0 (0.0)	0 (0.0)	0.0856
22F (n = 2)	2 (100.0)	0 (0.0)	0 (0.0)	0 (0.0)	0.0278
23A (n = 2)	0 (0.0)	0 (0.0)	2 (100.0)	0 (0.0)	0.7283
23B (n = 4)	3 (75.0)	0 (0.0)	1 (25.0)	0 (0.0)	0.0117
Sequence type^a^					
36 (n = 2)	2 (100.0)	0 (0.0)	0 (0.0)	0 (0.0)	0.0278
63 (n = 6)	0 (0.0)	0 (0.0)	6 (100.0)	0 (0.0)	0.0883
90 (n = 2)	0 (0.0)	0 (0.0)	2 (100.0)	0 (0.0)	0.7283
99 (n = 4)	0 (0.0)	0 (0.0)	0 (0.0)	4 (100.0)	< 0.0001
156 (n = 2)	0 (0.0)	2 (100.0)	0 (0.0)	0 (0.0)	0.2820
162 (n = 2)	0 (0.0)	0 (0.0)	2 (100.0)	0 (0.0)	0.7283
180 (n = 2)	0 (0.0)	0 (0.0)	2 (100.0)	0 (0.0)	0.7283
236 (n = 2)	0 (0.0)	2 (100.0)	0 (0.0)	0 (0.0)	0.2820
282 (n = 3)	0 (0.0)	3 (100.0)	0 (0.0)	0 (0.0)	0.0745
338 (n = 3)	0 (0.0)	0 (0.0)	3 (100.0)	0 (0.0)	0.5810
433 (n = 2)	2 (100.0)	0 (0.0)	0 (0.0)	0 (0.0)	0.0278
558 (n = 7)	1 (14.3)	6 (85.7)	0 (0.0)	0 (0.0)	0.0038
2331 (n = 3)	0 (0.0)	3 (100.0)	0 (0.0)	0 (0.0)	0.0745
2755 (n = 3)	0 (0.0)	0 (0.0)	3 (100.0)	0 (0.0)	0.5810
3111 (n = 3)	0 (0.0)	0 (0.0)	0 (0.0)	3 (100.0)	0.0009
5236 (n = 5)	0 (0.0)	0 (0.0)	5 (100.0)	0 (0.0)	0.1890

Data expressed as numbers (row percentages).

a Of the serogroups/serotypes determined as a single serogroup/serotype or determined sequence types, those which include two or more isolates are listed. See **Supplementary Table 2** for those which include only one isolate.

Among the isolates with identified STs, ST558 was the most frequent (seven isolates), followed by ST63 (six isolates) and ST5236 (five isolates). ST36, 99, 433, 558, and 3111 were significantly correlated with the presence of macrolide resistance genes (p < 0.05, respectively) (**Table 4**). Neither the ST36 nor ST433 isolates harbored macrolide resistance genes, whereas all ST99 and ST3111 isolates harbored both *mefA* and *ermB*. Of the seven ST558 isolates, six (85.7%) harbored *mefA* alone.

### Clinical features of patients with pneumococcal pneumonia

**Table 5** shows the clinical features of patients with pneumococcal pneumonia classified according to the presence of macrolide resistance genes in the isolates. Sex was significantly correlated with the presence of macrolide resistance genes. However, other variables, including classification of pneumonia, comorbidities, pneumonia severity, and mortality, showed no significant correlation with the presence of macrolide resistance genes in the isolates.

**Table 5:** Clinical features of 41 patients with pneumococcal pneumonia classified according to the presence of macrolide resistance genes in the isolates.

Variable	None (n = 5)	*mefA* (n = 13)	*ermB* (n = 18)	Both (n = 5)	P
Age (years)	56.0 (24.0)	65.0 (15.0)	68.5 (18.0)	67.0 (16.0)	0.3054
Sex (male/female)	4 (80.0)	3 (23.1)	13 (72.2)	4 (80.0)	0.0183
Classification					
CAP	3 (60.0)	8 (61.5)	10 (55.6)	2 (40.0)	0.9319
NHCAP	1 (20.0)	4 (30.8)	5 (27.8)	1 (20.0)	> 0.9999
HAP^a^	1 (20.0)	1 (7.7)	3 (16.7)	2 (40.0)	0.3238
Comorbidity					
Cerebrovascular/ neurological disease	2 (40.0)	0 (0.0)	4 (22.2)	0 (0.0)	0.0831
Cardiovascular disease	0 (0.0)	1 (7.7)	1 (5.6)	0 (0.0)	> 0.9999
Pulmonary disease	0 (0.0)	4 (30.8)	3 (16.7)	1 (20.0)	0.6483
Liver disease	0 (0.0)	1 (7.7)	0 (0.0)	1 (20.0)	0.2134
Renal disease	0 (0.0)	1 (7.7)	0 (0.0)	1 (20.0)	0.2134
Diabetes mellitus	0 (0.0)	2 (15.4)	2 (11.1)	0 (0.0)	> 0.9999
Collagen disease	1 (20.0)	1 (7.7)	1 (5.6)	2 (40.0)	0.1451
Malignancy	0 (0.0)	8 (61.5)	8 (44.4)	1 (20.0)	0.0855
A-DROP score, ≥ 3	1 (20.0)	0 (0.0)	2 (11.1)	0 (0.0)	0.3856
Positive of blood culture	0 (0.0)	1 (7.7)	4 (22.2)	0 (0.0)	0.4895
Initial antimicrobial therapy					
β-lactam alone	4 (80.0)	12 (92.3)	15 (83.3)	4 (80.0)	0.7921
β-lactam plus other classes	1 (20.0)	0 (0.0)	3 (16.7)	0 (0.0)	0.3338
Others	0 (0.0)	1 (7.7)	0 (0.0)	1 (20.0)	0.2134
30-day mortality	1 (20.0)	0 (0.0)	1 (5.6)	0 (0.0)	0.5280

Data expressed as median (interquartile range) or numbers (column percentages).

CAP, community-acquired pneumonia; NHCAP, nursing- and healthcare-associated pneumonia; HAP, hospital-acquired pneumonia.

a One patient with ventilator-associated pneumonia is included.

Among the three isolates from two patients with severe pneumonia (A-DROP score 3) and one patient with extremely severe pneumonia (A-DROP score 5), two harbored *ermB* alone and one no resistance genes. Their serogroups/serotypes were 3, 6C, and 22F. Among the five isolates from patients with bacteremia, four harbored *ermB* alone and one *mefA* alone. Their serogroups/serotypes were 3 (two isolates), 6C, 19A, and 13/28.

## Discussion

Our results demonstrated high rates of macrolide resistance, which was conferred by the presence of macrolide resistance genes. However, a novel ketolide, solithromycin, possessed potent *in vitro* activity, even in the presence of macrolide resistance genes. In addition, lascufloxacin, a novel fluoroquinolone, has demonstrated strong activity against pneumococcal isolates. Bacterial typing revealed the distribution of capsular and sequence types and clarified the relationships between the possession of macrolide resistance genes, serogroups/serotypes, and sequence types.

We observed a high macrolide resistance rate (86.3%) in this study, which is similar to previous studies from Japan, in which the resistance rate to erythromycin was 82.6% (Kawaguchiya et al., 2020) and only 2.6–8.1% and 2.1–4.8% showed susceptibility for clarithromycin and azithromycin, respectively (Okade et al., 2014). In this study, of the isolates that tested positive for *ermB*, including those harboring *ermB* alone and both *ermB* and *mefA*, 97.6% (41/42) showed higher MICs for macrolides (> 16 µg/mL) compared to isolates that tested positive for *mefA* alone (2–> 16 µg/mL). This confirmed previous reports that high- and intermediate-level resistance to erythromycin is conferred by *ermB*- mediated target modification and *mef*-mediated efflux, respectively (Lynch and Martinez, 2002; Xu et al., 2010). Additionally, the MICs for solithromycin were lower (0.03–0.25 μg/mL) than those for other macrolides, including against isolates harboring *mefA* and *ermB*. This result is consistent with previous reports showing that solithromycin is active against macrolide-resistant pneumococci, including those with *erm*- and *mef*-mediated resistance (McGhee et al., 2010). The activity of solithromycin against pneumococci harboring *erm* may be due to its ability to bind to ribosomes methylated by *erm* (Llano-Sotelo et al., 2010). However, although the MICs for solithromycin was low (0.03–0.25 µg/mL), 62.5% (5/8) of isolates with both *ermB* and *mefA* showed the highest MIC (0.25 µg/mL) (**Figure 3**, **Table 2**). Accumulation of macrolide resistance mechanisms might contribute to MIC elevation for solithromycin. In addition, a recent study reported novel mutations related to decreased susceptibility for solithromycin, including mutations in the intergenic regions of the macrolide resistance locus *mefE*/*mel* and in the vicinity of the ribosome binding site of *ermB* (Gingras et al., 2023). Because *S. pneumoniae* can acquire solithromycin resistance, surveillance of antimicrobial susceptibility for the drug should be continued.

The present study showed the frequencies of serogroups/serotypes and STs, and significant correlations between four serotypes (11A, 19A, 22F, and 23B) or five STs (ST36, 99, 433, 558, and 3111) and the presence of macrolide resistance genes. All ten isolates (100%) with serogroup 15 (serotypes 15A and 15B/C, five isolates each) possessed *ermB* alone, which was similar to a previous report from Japan describing a high prevalence (> 90%) for *ermB* in isolates with serotypes 15A, 15B, and 15C (Kawaguchiya et al., 2020). In this study, all four isolates (100%) with serotype 11A/ST99 harbored both *mefA* and *ermB* and no macrolide resistance genes were detected in any of the two isolates with serotype 22F/ST433. All three isolates (100%) with serotype 19A/ST3111 possessed both macrolide resistance genes, but all three isolates with serotype 19A/ST2331 harbored *mefA* alone.

In contrast to the high rate of macrolide resistance (86.3%), the rates of resistance to levofloxacin and moxifloxacin were only 2.7% (two isolates harboring *ermB*). However, their serogroup/serotype and ST were 13/28 and 338, respectively, indicating that specific serogroups/serotypes or sequence types might be related to fluoroquinolone resistance. In this study, the MICs for lascufloxacin tended to be lower than those for levofloxacin and moxifloxacin, and susceptibility for lascufloxacin was retained, even in isolates resistant to levofloxacin and moxifloxacin. Previous studies reported that substitutions of S79F in ParC and S81F in GyrA conferred high-level resistance to levofloxacin (MICs, 8–32 mg/L) and low-level resistance to moxifloxacin (MICs, 2–4 mg/L) (Weigel et al., 2001; Brueggemann et al., 2002), that were consistent with our results. Meanwhile, the MICs for lascufloxacin against levofloxacin- and moxifloxacin-resistant isolates were low (0.5 µg/mL) but highest among the isolates tested in this study. Therefore, we need continuous monitoring of antimicrobial susceptibility for this drug.

In our study, no significant differences were observed between clinical factors, excluding sex, and the presence of macrolide resistance genes in the isolates. All five bacteremia episodes were observed in patients with pneumonia caused by macrolide-resistant isolates (four with *ermB* alone and one with *mefA* alone) and their serogroups/serotypes were 3, 6C, 19A, or 13/28. A previous study, comparing hospitalized patients with pneumonia caused by macrolide-resistant isolates with those by macrolide-sensitive isolates, reported that the rate of bacteremia was significantly lower in patients with macrolide-resistant isolates. In this previous study, the rate of patients with pneumonia caused by macrolide-susceptible isolates was high (78.4%) compared to our study and serotypes of isolates that caused bacteremia were not shown (Cilloniz et al., 2015). Further studies are required to confirm the characteristics of macrolide-resistant *S. pneumoniae*, including pathogenicity, and the clinical features of patients with pneumonia caused by the bacteria.

This study has some limitations. The study was conducted in a single hospital in western Japan, which might not be representative of other regions or countries. The sample size was relatively small, potentially limiting the generalizability of the results. We did not investigate factors such as prior antibiotic use which might influence resistance patterns.

In conclusion, this study revealed the antimicrobial susceptibility, capsular type, and ST of the pneumococcal isolates obtained from our hospital. Macrolide resistance and possession of resistance genes were frequently observed, and specific capsular types and STs were correlated with the presence of macrolide resistance genes. *In vitro* activity of solithromycin and lascufloxacin against pneumococcal isolates was confirmed.

## Data availability statement

The sequences of MLST alleles and QRDR mutations are available in Nagasaki University's Acadamic Output Site (http://hdl.handle.net/10069/0002001041).

## Ethics statement

The studies involving humans were approved by the Institutional Review Board of Nagasaki University Hospital. The studies were conducted in accordance with the local legislation and institutional requirements. The ethics committee/institutional review board waived the requirement of written informed consent for participation from the participants or the participants’ legal guardians/next of kin because the study information was made public via the website and subjects were provided opportunity of refusal to participate in the study (opt-out method).

## Author contributions

TN: Investigation, Writing – original draft, Writing – review & editing. KK: Conceptualization, Supervision, Writing – original draft, Writing – review & editing, Project administration. NA: Methodology, Writing – review & editing. KO: Writing – review & editing. FM-K: Writing – review & editing. HH: Writing – review & editing. KI: Writing – review & editing. HM: Writing – review & editing. KY: Supervision, Writing – review & editing.
