# Supplementary material for: Molecular and phenotypic characterization of Streptococcus pneumoniae isolates in a Japanese tertiary care hospital
Source: Front Cell Infect Microbiol. 2024 Jul 22;14:1391879. doi: 10.3389/fcimb.2024.1391879 (PMC11298800; doi:10.3389/fcimb.2024.1391879)
Supplement: Supplementary file 1 [file Table_1.docx]

Supplementary Table 1. Primer sequences used in this study

| Target | Primer sequence | Reference |
| --- | --- | --- |
| *mefA* forward  *mefA* reverse  *ermB* forward  *ermB* reverse  *lytA* forward  *lytA* reverse  *perC* M0363  *parC* M4271  *gyrA* VGA3  *gyrA* VGA4 | 5′-GGGACCTGCCATTGGTGTGC-3′  5′-CCCAGCTTAGGTATACGTAC-3′  5′-CGTACCTTGGATATTCACCG-3′  5′-GTAAACAGTTGACGATATTCTCG-3′  5′-CAACCGTACAGAATGAAGCGG-3′  5′-TTATTCGTGCAATACTCGTGCG-3′  5′- TGGGTTGAAGCCGGTTCA-3′  5′- TGCTGGCAAGACCGTTGG-3′  5′-CCGTCGCATTCTTTACG-3′  5′- AGTTGCTCCATTAACCA-3′ | (NIID, 2021)  (NIID, 2021)  (NIID, 2021)  (NIID, 2021)  (NIID, 2021)  (NIID, 2021)  (Pan et al., 1996)  (Pan et al., 1996)  (Pan et al., 1996)  (Pan et al., 1996) |

Supplementary Table 2. Relationship between serogroup/serotype or sequence type and the presence of macrolide resistance genes

| Variable | None | *mefA* | *ermB* | Both | P |
| --- | --- | --- | --- | --- | --- |
| Serogroup/serotype^a^  7C (n = 1)  9V/A (n = 1)  18 (n = 1)  21 (n = 1)  33F (n = 1)  Sequence type^a^  166 (n = 1)  199 (n = 1)  355 (n = 1)  447 (n = 1)  717 (n = 1)  1233 (n = 1)  2758 (n = 1)  2771 (n = 1)  3116 (n = 1)  3117 (n = 1)  3594 (n = 1)  3787 (n = 1)  5242 (n = 1)  6183 (n = 1)  6686 (n = 1)  9396 (n = 1)  11184 (n = 1)  13347 (n = 1) | 0 (0.0)  0 (0.0)  1 (100.0)  0 (0.0)  0 (0.0)  0 (0.0)  0 (0.0)  1 (100.0)  1 (100.0)  0 (0.0)  0 (0.0)  0 (0.0)  0 (0.0)  0 (0.0)  0 (0.0)  1 (100.0)  0 (0.0)  0 (0.0)  0 (0.0)  0 (0.0)  0 (0.0)  1 (100.0)  0 (0.0) | 0 (0.0)  0 (0.0)  0 (0.0)  1 (100.0)  0 (0.0)  0 (0.0)  0 (0.0)  0 (0.0)  0 (0.0)  0 (0.0)  1 (100.0)  0 (0.0)  0 (0.0)  1 (100.0)  1 (100.0)  0 (0.0)  0 (0.0)  0 (0.0)  0 (0.0)  1 (100.0)  0 (0.0)  0 (0.0)  1 (100.0) | 1 (100.0)  1 (100.0)  0 (0.0)  0 (0.0)  0 (0.0)  1 (100.0)  1 (100.0)  0 (0.0)  0 (0.0)  0 (0.0)  0 (0.0)  1 (100.0)  1 (100.0)  0 (0.0)  0 (0.0)  0 (0.0)  1 (100.0)  1 (100.0)  1 (100.0)  0 (0.0)  1 (100.0)  0 (0.0)  0 (0.0) | 0 (0.0)  0 (0.0)  0 (0.0)  0 (0.0)  1 (100.0)  0 (0.0)  0 (0.0)  0 (0.0)  0 (0.0)  1 (100.0)  0 (0.0)  0 (0.0)  0 (0.0)  0 (0.0)  0 (0.0)  0 (0.0)  0 (0.0)  0 (0.0)  0 (0.0)  0 (0.0)  0 (0.0)  0 (0.0)  0 (0.0) | >0.9999  >0.9999  0.2466  0.5342  0.1096  >0.9999  >0.9999  0.2466  0.2466  0.1096  0.5342  >0.9999  >0.9999  0.5342  0.5342  0.2466  >0.9999  >0.9999  >0.9999  0.5342  >0.9999  0.2466  0.5342 |

Data expressed as numbers (row percentages)

^a^Of the serogroups/serotypes determined as a single serogroup/serotype or determined sequence types, those which include only one isolate are listed.
